# Water–Nitrogen Coupling Under Film Mulching Synergistically Enhances Soil Quality and Winter Wheat Yield by Restructuring Soil Microbial Co-Occurrence Networks

**DOI:** 10.3390/plants14223461

**Published:** 2025-11-13

**Authors:** Fangyuan Shen, Liangjun Fei, Youliang Peng, Yalin Gao

**Affiliations:** State Key Laboratory of Water Engineering Ecology and Environment in Arid Area, Xi’an University of Technology, Xi’an 710048, China; shenfangyaun@163.com (F.S.); pengyoul0927@163.com (Y.P.); g9122023@163.com (Y.G.)

**Keywords:** film mulching irrigation, water–nitrogen coupling, soil microbial networks, soil quality index, winter wheat yield, water and nitrogen use efficiency

## Abstract

Improper irrigation and fertilization can easily lead to soil nutrient imbalance, inhibit microbial reproduction, and thereby reduce soil quality and crop yield. This study conducted winter wheat planting experiments in 2023–2025, setting three muddy water (sediment-laden irrigation water) treatments of different sediment concentrations (3, 6 and 9 kg·m^−3^), irrigation levels (0.50–0.65, 0.65–0.80 and 0.80–0.95 FC), and nitrogen application rates (100, 160 and 220 kg·ha^−1^). An L9(3^3^) orthogonal experimental design was applied to evaluate the influence of water and nitrogen regulation on soil properties, microbial community structure, and wheat productivity. The results showed the following: Among these treatments, the T5 treatment (6 kg·m^−3^, 0.65–0.80 FC, 160 kg·ha^−1^) significantly improved the root zone environment, and the total nitrogen (TN), ammonium nitrogen (NH_4_^+^-N), nitrate nitrogen (NO_3_^−^-N), and soil organic carbon (SOC) content also increased significantly. T5 also enhanced the diversity and network complexity of bacterial and fungal communities. Notably, genera such as *Lysobacter*, *Lasiobolidium*, and *Ascobolus* became central to nitrogen transformation and nutrient cycling. Structural equation modeling revealed the interdependent mechanism between soil quality, microorganisms, and wheat yield: NO_3_^−^-N and SOC drive improvements in soil quality, while microbial community structure and network complexity are key to yield increases, with fungal communities making the largest direct contribution to yield (*R*^2^ = 0.93). The T5 treatment increased two-year yields by 21.34–24.96% compared to conventional irrigation and fertilization (CK2), improved irrigation water use efficiency by 56.40–57.51% and peak nitrogen agronomic efficiency. The synergistic effect of “soil quality optimization–enhanced microbial activity–efficient utilization of water and nitrogen–high wheat yield” has been achieved, providing a theoretical basis and practical reference for scientific water and nitrogen management and sustainable yield increase in winter wheat in the Yellow River Basin and similar areas.

## 1. Introduction

Food security is intrinsically linked to human well-being and acts as a cornerstone of global socioeconomic stability [1]. To meet the rising global food demand, intensive agricultural practices have been widely adopted [2]. However, the overuse of irrigation and fertilization has led to resource waste, soil quality degradation, and environmental pollution [3]. These problems contribute to a decline in soil health and crop productivity, posing a serious threat to the long-term sustainability of agricultural systems. Wheat, one of the most widely consumed cereal crops worldwide and a staple crop in China, plays a key role in securing food availability and ensuring social and economic stability [4]. However, in Chinese wheat production systems, excessive fertilizer inputs and poorly regulated irrigation practices have intensified risks of soil degradation and environmental damage [5]. There is an urgent need to develop scientifically sound irrigation and fertilization strategies that enhance crop yields, restore soil quality, and promote sustainable agricultural development [6].

Soil quality forms the basis of agricultural productivity and ecosystem functionality [7] and is determined by key indicators such as physical, chemical, and biological properties [8]. Among these, nutrient concentrations including nitrogen (N), phosphorus (P), potassium (K), and soil organic carbon (SOC) as well as soil structural stability and microbial community characteristics, exert a critical influence on overall soil health [9]. Moreover, research indicates that optimizing nutrient inputs and outputs through irrigation and nitrogen management can sustainably improve soil quality and fertility [10]. Soil microorganisms serve as key drivers of soil ecosystem processes. They mediate nutrient cycling, organic carbon decomposition, and pollutant breakdown [11]. The diversity and network complexity of soil microbial communities are considered reliable biological indicators of soil quality [12]. Due to their high sensitivity to environmental conditions, microbial communities can rapidly reflect dynamic shifts in soil status [13]. Therefore, understanding microbial responses to agronomic interventions is essential for improving soil quality and crop productivity. The diverse responses of soil properties and microbial communities to irrigation and fertilization complicate accurate assessment of their combined impact on soil quality and yield outcomes [14]. In this context, the soil quality index (SQI) has received increasing attention for its integrative capacity, multifunctionality, and flexibility in evaluating soil conditions [15]. Applying SQI to evaluate different irrigation and fertilization strategies offers an effective framework for guiding agricultural management [16]. Improvements in soil quality and microbial activity have been shown to significantly boost crop performance [17]. Yang et al. [18] identified soil quality and functional microbial communities as primary drivers of increased yields. Similarly, Zhang et al. [19] demonstrated that optimized nitrogen inputs and appropriate soil moisture enhanced root-zone microbial activity, biochemical properties, and wheat grain yield in the Huanghuai Plain. Moderate irrigation was identified as the optimal approach for achieving stable and high yields.

In recent years, plastic film mulching has been widely adopted in arid and semi-arid farmlands of northwestern China due to its capacity to raise soil temperature [20], suppress evaporation in the root zone, inhibit weed growth, and improve crop productivity as well as water-nitrogen use efficiency [21,22]. This technology also enhances soil quality by modifying its physical, chemical, and biological properties [23]. Li et al. [24] reported that, compared to the control (CK), film mulching significantly improved soil fertility and biological activity, increasing the soil quality index (SQI) by 7.2%. Film-hole irrigation technology, which integrates the benefits of plastic mulching and precision irrigation, effectively regulates the soil hydrothermal environment and significantly boosts water use efficiency and crop yields [25,26]. In the arid and semi-arid regions of northwest China, water resources are severely limited [27]. To alleviate irrigation water shortages, muddy water (here defined as sediment-laden irrigation water) is commonly used in agricultural areas along the Yellow River Basin [28], making it a distinctive regional practice. Long-term studies have demonstrated that soil irrigated with muddy water supports healthy crop growth and results in sustained improvements in both soil quality and yields over time [29].

Although previous studies have examined the effects of irrigation and nitrogen application on crop yield or individual soil variables from various perspectives, a systematic understanding of the multi-level coupled mechanism linking “muddy water irrigation–soil physicochemical properties–microbial communities–crop yield” remains limited, particularly under the muddy water film hole irrigation (water-nitrogen synergy + film hole irrigation) system. the driving pathways through which microbial community diversity influences soil quality and yield formation have not been thoroughly elucidated. Therefore, this study, based on the unique agricultural background of the arid and semi-arid regions of the Yellow River Basin, designed an L9(3^3^)orthogonal experiment to systematically assess the synergistic effects of different combinations of muddy water silt content, irrigation levels, and nitrogen application rates on winter wheat rhizosphere soil nutrient characteristics, microbial community diversity and network complexity, soil quality index (SQI), and yield. Additionally, using structural equation modeling (PLS-SEM), to construct a “soil–microbe–crop” multi-pathway regulatory framework, elucidating the ecological functional roles of key microbial groups in water-nitrogen responses and yield formation. This study is the first to reveal, from a systems ecology perspective, the interactive mechanisms of water-nitrogen management on the “soil–microbe–crop” system under muddy water film hole irrigation conditions. It clarifies that water-nitrogen regulation can simultaneously optimize soil quality and water-nitrogen use efficiency while enhancing yield, providing theoretical support and practical guidance for scientific water-nitrogen management and sustainable winter wheat yield increases in the Yellow River Basin and similar ecological regions.

## 2. Materials and Methods

### 2.1. Experimental Site

The field experiment was carried out under a rainproof shelter at the open-air experimental station affiliated with the Department of Modern Agricultural Engineering, Xi’an University of Technology (34°15′ N, 108°59′ E; elevation: 447 m). The site experiences an average annual temperature of 12.9 °C, with total yearly precipitation reaching 612.1 mm. Long-term average potential evaporation is approximately 1440 mm, annual sunshine duration accumulates to 2164 h, and the frost-free period spans roughly 221 days. Winter wheat is typically sown in mid-October and harvested by the end of the following May. Meteorological parameters, including daily maxima and minima of air temperature and precipitation dynamics, were recorded using a Davis automatic microclimate station installed at the site. Detailed weather trends during the experimental period are illustrated in Supplementary S1.

The experimental soil was classified as a silt loam. According to the IUSS Working Group WRB (2022) [30], the soil is classified as an Anthrosol, reflecting the long-term, intensive agricultural use and sediment-rich irrigation practices characteristic of the region. The particle size distribution was 14.87% sand (0.05–2 mm), 71.55% silt (0.002–0.05 mm), and 13.58% clay (<0.002 mm). The soil at the experimental site had a bulk density of 1.30 g cm^−3^ and a field capacity of 23.50% (gravimetric basis). Within the 0–60 cm soil profile, the mean physicochemical properties were as follows: soil organic carbon 9.14 g·kg^−1^, total nitrogen 0.85 g·kg^−1^, nitrate nitrogen 7.28 mg·kg^−1^, ammonium nitrogen 22.20 mg·kg^−1^, available potassium 128 mg·kg^−1^, available phosphorus 21.40 mg kg^−1^, and pH 7.61. Each plot covered an area of 12 m^2^ (5 m × 2.4 m). A transparent polyethylene shelter, transmitting more than 90% of photosynthetically active radiation (PAR), was installed to exclude rainfall while permitting full sunlight exposure. To maintain near-natural atmospheric conditions, the sidewalls remained fully open during the entire trial, ensuring adequate ventilation and minimizing microclimatic deviation from surrounding farmland. The shelter was only deployed during precipitation events to prevent uncontrolled irrigation. Outside of these periods, the plots experienced radiation, wind, and temperature similar to the adjacent open fields. A 2 m buffer strip was established around each plot to mitigate edge effects, and all observations were confined to the central protected area.

### 2.2. Experimental Design

The winter wheat variety ‘Xinong 575’ was used as the test crop in this study. Before sowing in mid-October, pre-irrigation was applied to ensure that the 0–60 cm soil profile reached the target moisture thresholds specified for each treatment. Following land leveling, experimental plots were entirely covered with polyethylene mulch film measuring 1.2 m in width and 0.008 mm in thickness. The film edges were secured by upward folding and soil pressing to prevent water ingress along the seams. Planting ridges were constructed at 45 cm intervals, with each ridge 20 cm in height and width. Time domain reflectometry (TDR) probes were installed both between ridges and atop them to monitor volumetric soil water content. Utilizing the film-hole irrigation approach, 7 cm-diameter planting holes were perforated through the mulch layer at regular intervals. The center-to-center spacing of the film holes was 15 cm, with the same row spacing, and seeds were sown to a depth of 5 cm (Supplementary S2). Hole sowing was employed, with 10–15 seeds placed per hole; after germination, plants were thinned to eight per hole, establishing a final population density of approximately 2.87 million plants per hectare. Irrigation water was applied in furrows between ridges, allowing muddy water to flow across the surface of the plastic mulch. Fine sediment particles infiltrated the root zone via the film holes, while coarser sediments were deposited atop the mulch as flow continued. With increasing sediment concentration and irrigation frequency, the thickness of deposited sediment layers varied, thereby influencing the thermal regime of the soil beneath the mulch.

To simulate the sediment concentration typical of water irrigation in the Yellow River’s middle and upper reaches, this study was based on the measured annual sediment concentration range of the basin (2.9 to 13.5 kg·m^−3^). The chemical composition of the sediment in the sediment-laden irrigation water was as follows: pH 7.6, soil organic carbon (SOC) 8.35 g·kg^−1^, total nitrogen (TN) 0.72 g·kg^−1^, total phosphorus (TP) 1.38 g·kg^−1^, total potassium (TK) 19.6 g·kg^−1^, available phosphorus 18.2 mg·kg^−1^, and available potassium 56.41 mg·kg^−1^. To ensure the experiment accurately reflects real-world agricultural conditions involving sandy water irrigation, this study established three levels of sediment concentration: ρ1 (3 kg·m^−3^), ρ2 (6 kg·m^−3^), and ρ3 (9 kg·m^−3^). Concurrently, the study established three different irrigation levels based on field capacity (FC, 23.5%): W1 (0.50–0.65 FC), W2 (0.65–0.80 FC), and W3 (0.80–0.95 FC), where irrigation was initiated when soil moisture fell below the lower threshold of each treatment and terminated upon reaching the upper boundary. Furthermore, the experiment included three graded nitrogen application treatments: N1 (100 kg·ha^−1^), N2 (160 kg·ha^−1^), and N3 (220 kg·ha^−1^). The core of this study was a systematic L9(3^3^) orthogonal experimental design, which is a statistically robust method for evaluating the main effects of three factors at three levels each (Sediment: ρ1–3; Irrigation: W1–3; Nitrogen: N1–3). This design generated the nine primary treatment combinations (T1–T9) shown in Table 1. Separately, to benchmark these factorial treatments against zero-nitrogen and conventional practices, two additional reference controls were included: CK1: clean water irrigation, irrigation (0.80–0.95 FC), no nitrogen input; and CK2: clean water irrigation, irrigation (0.80–0.95 FC), 220 kg·ha^−1^ nitrogen. This experimental layout comprised 11 treatments in total, each replicated three times. Fertilizers included urea (46% N), calcium superphosphate (6.98% P), and potassium sulfate (42.32% K). Prior to sowing, 60% of the total nitrogen dose for each respective treatment, along with full doses of phosphorus (163 kg P·ha^−1^) and potassium (115 kg K·ha^−1^), were incorporated into the soil at a 15 cm depth. The remaining 40% of the nitrogen dose was broadcast at the regreening stage. A full summary of treatment arrangements is presented in Table 1.

### 2.3. Sample Collection and Measurements

#### 2.3.1. Soil Sample Collection

Soil sampling was conducted at the winter wheat maturity stage (e.g., late May) in growing seasons, immediately before the crop harvest. soil samples (0–20 cm) were collected using an S-shaped sampling strategy, with five subsamples obtained per treatment. After promptly removing roots, weeds, stones, and other debris, the soil was passed through a 1 mm sieve. Subsamples from the same plot were thoroughly homogenized into a composite sample, sealed in clean self-locking polyethylene bags, and appropriately labeled. Fresh samples were rapidly immersed in liquid nitrogen and transported to the laboratory under cryogenic conditions. Each composite sample was divided into three aliquots for different analytical purposes. One portion was stored at −80 °C for subsequent DNA extraction and microbial community structure and function analysis. Another was kept at 4 °C for assessment of available nutrient contents. The third portion was air-dried for evaluating basic soil physicochemical properties, including nutrient content and pH.

#### 2.3.2. Soil Physicochemical Property Determination

Soil particle size distribution was determined by the hydrometer method. Soil bulk density was measured using the core sampler method and field capacity was determined by the pressure plate method (−33 kPa). Available phosphorus (AP) was extracted using the 0.5 mol·L^−1^ NaHCO_3_ (Olsen) method and determined colorimetrically. Available potassium (AK) was extracted with 1.0 mol·L^−1^ NH_4_OAc and measured by flame photometry. Soil pH was measured using a pH meter (*FE20 Five Easy™* Mettler Toledo, Columbus, OH, USA) with a soil-to-water ratio of 1:2.5 [31]. Organic carbon content was determined Via dichromate oxidation with concentrated H_2_SO_4_ and back-titration using FeSO_4_ [32]. Total nitrogen was quantified using the Kjeldahl digestion method followed by automated detection [33]. NH_4_^+^-N and NO_3_^−^-N concentrations were assessed by continuous flow analysis after extraction with 2 mol·L^−1^ KCl [34]. All measurements were conducted in triplicate, and the mean values were used for analysis. Soil temperature was continuously recorded with a temperature probe (*179-DT*, Apresys, Shanghai, China) installed beneath the mulch at 5, 10, 15, and 20 cm depths. Soil water content was determined using a portable moisture meter (*TRIME-PICO* tubular time domain reflectometry (TDR) system, IMKO Micromodultechnik GmbH, Ettlingen, Germany). Measurements were taken across the 0–100 cm soil profile at 10 cm intervals. Monitoring was conducted at three-day intervals, with additional measurements taken immediately before and after irrigation events to capture short-term changes in soil water status. All the measurement data from the two years were sorted out, and the average value was calculated for subsequent analysis.

#### 2.3.3. Collection and Determination of Winter Wheat Plant Samples

Plant height: Plant height was measured at the critical growth stage of winter wheat. A representative number of 10 winter wheat plants was selected from each plot using the “fixed plant measurement” method. Using a tape measure, measure the vertical distance from the base of the stem (soil surface) to the highest point of the top of the plant (e.g., the top of the spike or the apex of the tallest leaf in its naturally extended state). Repeat the measurements for the same batch of labeled plants at the same location to track their dynamics.

Dry matter: Dry matter measurements were made at the key growth stage of winter wheat. Ten winter wheat plants with uniform growth were randomly selected in each test plot, and the plants were cut down along the soil surface with scissors to remove surface dirt, and divided into three parts: leaves, stems, and spikes. The plants were bagged according to each organ and put into an oven at 105 °C for 30 min, then baked at 75 °C until constant weight, and the dry mass was weighed by an electronic balance with a precision of 0.01 g.

Yield: At the maturity of winter wheat, 20 winter wheat plants were selected from each plot to count the number of grains per spike, and three 1 m^2^ sample areas were taken to investigate the effective number of spikes, threshing and drying to determine the seed yield, and then 1000 grains were taken from the harvested grains of each plot to determine the thousand-grain weight.

#### 2.3.4. DNA Extraction and Bioinformatics Analysis

Fresh soil samples were submitted to Novogene Co., Ltd. (Beijing, China) for microbial community analysis Via amplicon sequencing of bacterial 16S rRNA (V3–V4) and fungal ITS1 regions. Genomic DNA was extracted using the CTAB method [35], and its quality was evaluated by spectrophotometry, agarose gel electrophoresis, and Bioanalyzer (Agilent, Santa Clara, CA, USA). Bacterial and fungal communities were amplified using primers 341F/806R and ITS5-1737F/ITS2-2043R, respectively. PCR products were visualized by agarose gel, purified with magnetic beads, and pooled for library construction using the *TruSeq^®^* DNA PCR-Free Kit (Illumina, San Diego, CA, USA). Libraries were sequenced on the Illumina *NovaSeq* 6000 platform with 2 × 250 bp reads. Raw data processing included barcode removal (Cutadapt v3.3), read merging *FLASH* [36], and quality filtering (*fastp*). High-quality reads were denoised and clustered into ASVs using *DADA2 in QIIME2 (v2022.2)*. Chimeras were removed by aligning sequences against *SILVA* (for bacteria) and *UNITE* (for fungi) databases using *VSEARCH* [37]. Taxonomic assignment was performed using *QIIME2’s* q2-feature-classifier (confidence = 0.7). To reduce sequencing bias, ASV tables were rarefied (48,249 reads for 16S; 69,708 for ITS). Relative abundances were calculated from the normalized data. Alpha diversity (Shannon, Simpson, Chao1, observed features) and beta diversity (Bray–Curtis, UniFrac distances) were analyzed to compare microbial community composition. NMDS, PCoA, and PCA were used for ordination analysis. All treatments were conducted in biological triplicate, and analysis followed Novogene’s standardized protocols [38].

### 2.4. Soil Quality Index and Water and Nitrogen Use Efficiency of Winter Wheat

#### 2.4.1. Winter Wheat Harvest Index and Water and Nitrogen Use Efficiency

(1) Irrigation water use efficiency (*IWUE*, kg·m^−3^) [39]

*IWUE* = *Y*/*I* (1)

where *Y* is winter wheat yield (kg·ha^−1^), *I* is irrigation water (m^−3^·ha^−1^).

(2) Agronomic efficiency of nitrogen (*AE_N_*, kg·kg^−1^) [40]

*AE_N_* = (*Y_T_* − *Y*_0_)/*F_N_* (2)

where *Y_T_* is the yield in plots where nitrogen was applied (kg·ha^−1^); *Y*_0_ is the yield in plots where no nitrogen was applied (kg·ha^−1^). *F_N_* is the amount of nitrogen fertilizer applied in the treatment plot (kg·ha^−1^). In this study, CK1 treatment was used as a unified zero nitrogen baseline yield (*Y*_0_) representing local conventional practices. The purpose of using this unified baseline is to evaluate and relatively compare the nitrogen fertilizer yield increase efficiency of each water nitrogen sand coupling treatment (T1–T9) in the L9(3^3^) orthogonal experiment compared to the conventional farmer model.

(3) Harvest index (*HI*) [41]

(3) HI=Y/DM

where DM is the winter wheat dry matter accumulation (kg·ha^−1^).

#### 2.4.2. Soil Quality Evaluation

The soil quality index (SQI) is a metric used to evaluate soil quality, with higher values indicating better soil conditions in the root zone of wheat [42]. Seven soil indicators, NH_4_^+^-N, NO_3_^−^-N, SMC, pH, TN, SOC, and T, were selected to characterize the soil environment. These indicators were standardized to dimensionless values using an affiliation function, and the affiliation function value for each indicator was calculated. For indicators positively correlated with soil quality, Equation (4) was applied, while Equation (5) was used for those negatively correlated.

(4) $$F\left(X_{\mathrm{ij}}\right)=\frac{\left(X_{\mathrm{ij}}-X_{\min}\right)}{\left(X_{\max}-X_{\min}\right)}$$

(5) $$F\left(X_{\mathrm{ij}}\right)=1-\frac{\left(X_{i}-X_{\min}\right)}{\left(X_{\max}-X_{\min}\right)}$$

where $X_{\mathrm{ij}}$ is the jth indicator in the ith treatment; $X_{\max}$ and $X_{\min}$ are the maximum and minimum values of the jth indicator under all treatments, respectively; and $F\left(X_{\mathrm{ij}}\right)$ is the value of the affiliation function of the jth indicator in the *i*th treatment.

Following the method described by Mazzon et al. [43], SPSS 21.0 was used to perform factor analysis on the selected soil indicators. This analysis provided the common factor variance for each indicator, which reflects its contribution to the quality of the soil micro-ecological environment. The weight of each indicator was calculated as the percentage of its common factor variance relative to the total common factor variance. Finally, the SQI values for different irrigation and fertilization treatments were determined using Equation (6).

(6) $$\;\mathrm{SQI}=\sum_{i=1}^{n}\;\sum_{j=1}^{n}\;F\left(X_{\mathrm{ij}}\right)\times W\left(X_{\mathrm{ij}}\right)$$

where $W\left(X_{\mathrm{ij}}\right)$ is the weight of the j th indicator of the ith treatment and n is a positive integer.

#### 2.4.3. Calculation of the Co-Occurrence Network

Microbial symbiotic networks were constructed and analyzed using the molecular symbiotic network analysis pipeline [44]. Symbiotic network analyses for bacteria and fungi at the genus level were performed based on Spearman correlation coefficients using the psych package in *R4.1.0* [45]. Only species with strong positive or negative correlations (Spearman correlation coefficient r > 0.7) and statistically significant correlations (*p* < 0.05) were retained. To avoid bias, only species greater than 60% of the sample size were retained for analysis. Correlation networks were constructed in *Gephi 0.10.1* software, where each node represents an ASV (Amplicon Sequence Variant), and edges represent correlations between nodes. Network topology metrics, including the number of nodes, number of edges, network density, clustering coefficient, and degree of the co-occurrence network, were calculated using Gephi’s Analyzer tool. Node roles within the symbiotic networks were characterized by intra-module connectivity (Zi) and inter-module connectivity (Pi) [46] Nodes were categorized into four topological roles based on Zi and Pi thresholds: network hubs (Zi > 2.5; Pi > 0.62), module hubs (Zi > 2.5; Pi < 0.62), connectors (Zi < 2.5; Pi > 0.62), and peripherals (Zi < 2.5; Pi < 0.62) [47]. Since symbiotic network topology parameters (e.g., number of edges, number of nodes, average degree, connection density, and connectivity) reflect network complexity, bacterial and fungal network topology metrics were used to represent their respective network complexities [48].

### 2.5. Statistical Analysis

Microsoft Excel 2016 was utilized to calculate the mean and standard deviation of the raw data. One-way ANOVA was performed on the soil physicochemical indices of winter wheat using *SPSS 21.0* software. Differences in soil microbial Alpha diversity index and microbial community abundance among different water nitrogen treatments were analyzed using the NovoMagic Microbial Online data processing platform (https://magic.novogene.com (accessed on 23 May 2025)) and the test of variance (ANOVA). Linear mixed-effects models were constructed in *R* (*version 4.2.2*) using the “*lme4*” package to analyze the relationships between key soil physicochemical indices, microbial diversity indices, microbial community indices, and microbial network complexity indices with soil quality (SQI) and yield. Partial least squares structural equation modeling (PLS-PM) [49] was also combined to analyze the direct and indirect effects of management practices (ρ, W, N), soil quality, microbial community and microbial network complexity indicators on winter wheat yield. Mapping was performed using Origin 2020b software.

## 3. Results

### 3.1. Physicochemical Properties of Winter Wheat Soils

**Figure 1:**
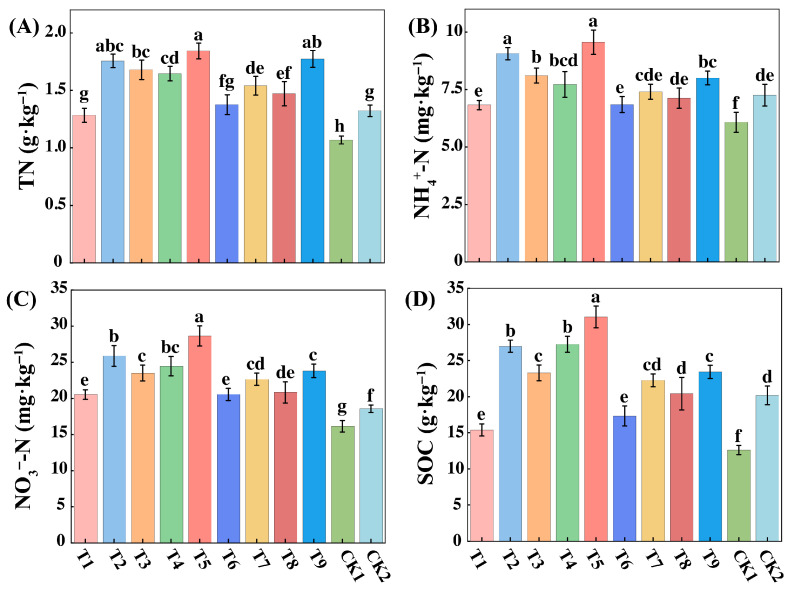
Physical and chemical properties of winter wheat soil. Note: TN (**A**) denotes total nitrogen (g·kg^−1^), NH_4_^+^-N (**B**) denotes ammonium nitrogen (mg·kg^−1^), NO_3_^−^-N (**C**) denotes nitrate nitrogen (mg·kg^−1^), SOC (**D**) denotes organic carbon (g·kg^−1^). Different lowercase letters denote significant differences at *p* < 0.05. the same below.

Soil nutrient levels varied significantly across the different treatments, with the T5 treatment consistently demonstrating the most substantial improvements in key indicators (Figure 1). Specifically, the total nitrogen (TN) content in the T5 treatment was the highest observed, representing a 72.43% and 39.31% increase compared to the CK1 and CK2 control groups, respectively (Figure 1A). A similar and pronounced trend was observed for mineral nitrogen. The ammonium nitrogen (NH_4_^+^-N) content in T5 was 31.73% higher than in the CK2 control (Figure 1B). Furthermore, the nitrate nitrogen (NO_3_^−^-N) level in T5 was 77% higher than in the CK1 control (Figure 1C). The T5 treatment also proved most effective at enhancing soil organic carbon (SOC), reaching a level of 31.03 g/kg, which was 53.73% higher than the CK2 controls, respectively (Figure 1D). Other soil properties were also affected by the treatments (Supplementary S3A–D). The highest soil moisture content (SMC) was recorded in the T6 treatment. Concurrently, soil temperature (T) showed a decreasing trend with higher sediment concentrations, reaching its lowest point in the T9 treatment (22.23 °C). Corroborating the individual nutrient results, the comprehensive soil quality index (SQI) also peaked in the T5 treatment (0.68), confirming it as the most effective strategy for improving overall soil quality.

### 3.2. Soil Microbial Community and Network Co-Occurrence of Winter Wheat

#### 3.2.1. Microbial Diversity of Winter Wheat

The diversity indices of soil bacteria and fungi in winter wheat varied significantly among different water and nitrogen treatments (Table 2). The alpha diversity indices of microbial communities exhibited a trend of initially increasing and then decreasing as the levels of muddy water sediment concentration, irrigation, and nitrogen application increased. The Chao1, ACE, and Richness indices of soil bacteria reached their highest values under the T5 treatment, with the Chao1 index increasing by 53.39% and 48.22% compared to CK1 and CK2, respectively. For the fungal community, the T5 treatment fostered the highest overall diversity (Shannon and Simpson indices) and observed richness (Richness index). Notably, the Chao1 and ACE indices peaked under the T2 treatment, indicating a distinct response of the fungal community to high nitrogen application under low sediment conditions. In summary, while the T5 treatment demonstrated the best overall balance of microbial community diversity, the T2 treatment was specifically favorable for maximizing the estimated richness of fungi.

#### 3.2.2. Microbial Community of Winter Wheat

**Figure 2:**
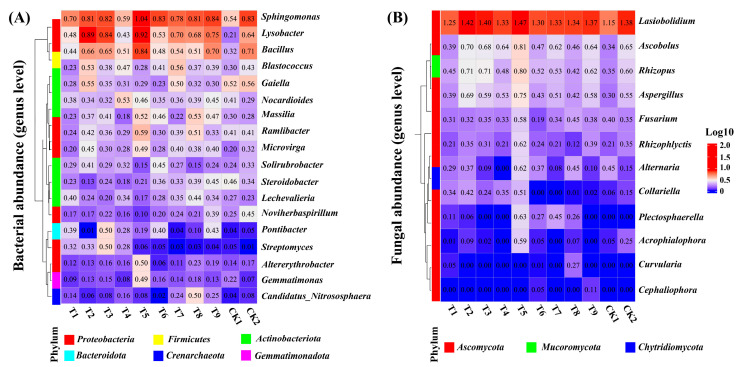
Winter wheat microbial community abundance (genus level). Note: The clustered heatmap (**A**,**B**) demonstrates the abundance of soil bacteria and fungi at the genus level.

Different water and nitrogen treatments significantly influenced the structure of soil bacterial communities (Figure 2A). Among these, *Sphingomonas* and *Lysobacter* were the dominant genera, with a combined relative abundance exceeding 8%. *Sphingomonas* reached its highest abundance (10.02%) under the T5 treatment, while *Lysobacter*, Bacillus, and *Blastococcus*, capable of mineralizing organic nitrogen, also peaked under the same treatment. In fungal communities (Figure 2B), *Lasiobolidium* and *Ascobolus*, associated with nitrogen cycling, showed significantly higher abundances in the T5 treatment, reaching 28.21% and 5.41%, thereby promoting organic matter decomposition and nitrogen release. Supplementary S4A–D further demonstrates that *Rhizopus* abundance exceeded 4% in both the T2 and T3 treatments with low sediment concentration, suggesting its potential role in nitrogen transformation under low-sand conditions. The CK1 control treatment exhibited significantly lower abundances of dominant genera compared with the other treatments. Compared with conventional irrigation and fertilization (CK2), the abundances of *Sphingomonas* and *Lasiobolidium* increased by 71.94% and 21.98%, respectively, under the T5 treatment.

#### 3.2.3. Soil Microbial Co-Occurrence Networks of Winter Wheat

**Figure 3:**
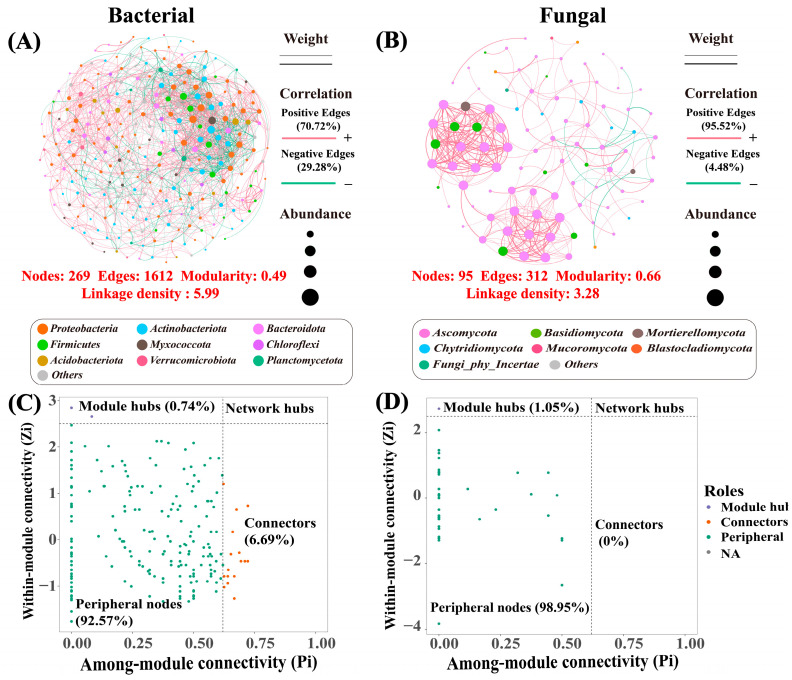
Soil microbial symbiotic network of winter wheat. Note: Visualization of bacterial (**A**) and fungal (**B**) co-occurrence networks in winter wheat soil under muddy water film hole irrigation water nitrogen regulation. The size of the nodes is proportional to the relative abundance of the ASVs, and the color of the nodes indicates different bacterial taxa. The width of each edge is proportional to Spearman’s correlation coefficient, and red and green edges indicate positive and negative relationships, respectively. (**C**,**D**) are plots of the distribution of keystone taxa in this winter wheat soil bacteria and fungi in four subcategories: network hubs (Zi > 2.5; Pi > 0.62), module hubs (Zi > 2.5; Pi < 0.62), connectors (Zi < 2.5; Pi > 0.62), and peripherals (Zi < 2.5; Pi < 0.62).

Different water and nitrogen treatments significantly influenced the topological characteristics of bacterial (Figure 3A) and fungal (Figure 3B) microbial co-occurrence networks in winter wheat soil. The bacterial network contained 269 nodes and 1612 edges, with a modularity coefficient of 0.49 and a link density of 5.99, while the fungal network contained 95 nodes and 312 edges, with a modularity coefficient of 0.66 and a link density of 3.28. The bacterial network was more complex, with significant competition, while the fungal network exhibited higher modularity and co-occurrence. The bacterial network had 92.57% peripheral nodes and 6.69% connected nodes, indicating high inter-modular connectivity (Pi > 0.62, Figure 3C). The fungal network had 98.95% peripheral nodes and only 1.05% core nodes, indicating a lack of bridge nodes across modules (Figure 3D). This indicates that the roles and network structures of bacteria and fungi in soil microbial communities were significantly different, with bacteria exhibiting more cross-module synergistic properties. Supplementary S5A–D shows that different muddy water sediment concentration, irrigation, and nitrogen application significantly affected network topology. Moderate sand–water–nitrogen inputs (ρ2, W2, N2) improved network stability and interactions among microbial and maintained the structure and function of microbial networks. In contrast, extreme water-nitrogen conditions (e.g., ρ1, W3, N3) weakened the microbial co-occurrence network and may negatively impact the health and stability of the soil ecosystem.

### 3.3. Plant Height and Dry Matter of Winter Wheat

**Figure 4:**
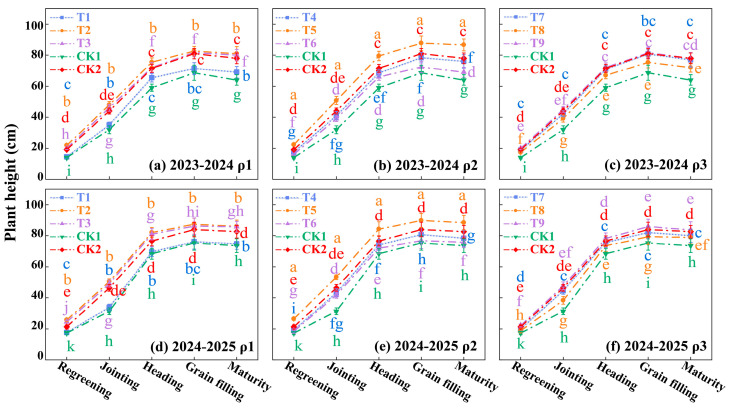
Plant height of winter wheat in 2023–2024 and 2024–2025. Note: ρ1, 3 kg·m^−3^ sediment concentration; ρ2, 6 kg·m^−3^ sediment concentration; ρ3, 9 kg·m^−3^ sediment concentration; CK1 refers to the treatment with clean water irrigation, full irrigation (0.80–0.95 FC), no nitrogen application; CK2 refers to the treatment with clean water irrigation, full irrigation (0.80–0.95 FC), 220 kg·ha^−1^ nitrogen application. The data are the average values from three parallel treatments for plant height of winter wheat in 2023–2024 and 2024–2025. Different lowercase letters denote significant differences at *p* < 0.05. The same below.

**Figure 5:**
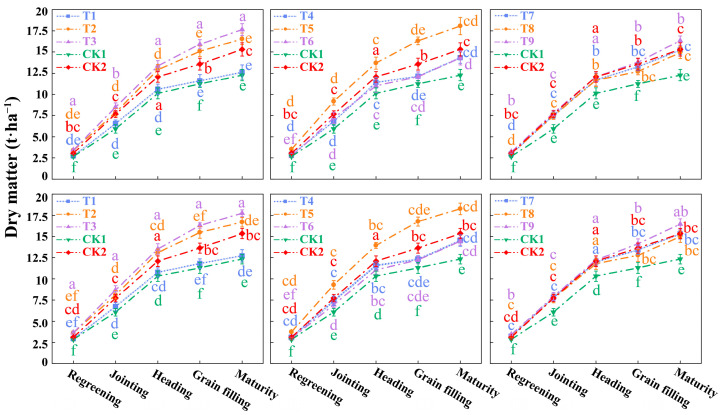
Dry matter of winter wheat in 2023–2024 and 2024–2025. Note: The data are the average values from three parallel treatments for dry matter of winter wheat. Different lowercase letters denote significant differences at *p* < 0.05.

As shown in Figure 4 and Figure 5, plant height and dry matter accumulation in both growing seasons exhibited a similar “slow-fast-slow” dynamic, peaking at the maturity stage. Analysis of the orthogonal treatments (T1–T9) reveals a strong and consistent advantage for moderate (Level 2) inputs of sediment, water, and nitrogen. T2 treatment (moderate water, W2) and T3 treatment (moderate nitrogen, N2), showed relatively high performance. However, the optimal result was achieved only when all three factors were combined at their moderate levels. The T5 (ρ2W2N2) treatment consistently and significantly outperformed all other combinations, including those with only partial moderate inputs. Compared to the conventional irrigation and fertilization CK2 treatment, plant height under the T5 treatment increased by 8.23% and 6.92%, while dry matter increased by 17.98% and 19.00% in the respective years. This demonstrates that the synergistic combination of moderate sediment, moderate water, and moderate nitrogen is the optimal strategy. Analysis of variance (ANOVA) confirmed that all three factors (ρ, W, N) had significant effects (*p* < 0.05) on both plant height and dry matter, with nitrogen application (N) generally showing the most pronounced effect (*p* < 0.01).

### 3.4. Yield and Water and Nitrogen Utilization Efficiency of Winter Wheat

**Figure 6:**
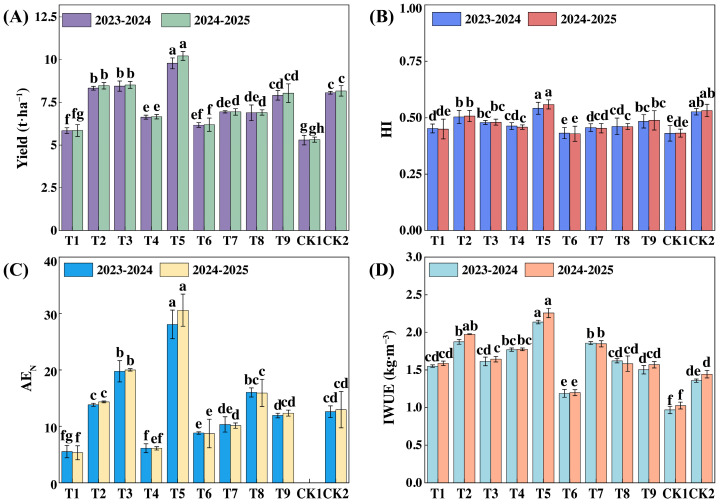
Winter wheat yield and water and nitrogen use efficiency in 2023–2024 and 2024–2025. Note: The data are average values from three parallel treatments for the yield (**A**), harvest index (**B**), agronomic efficiency of nitrogen (**C**), and irrigation water use efficiency (**D**) of s winter wheat in 2023–2024 and 2024–2025. HI, harvest index; AE_N_, agronomic efficiency of N (kg·kg^−1^); IWUE, irrigation water use efficiency (kg·m^−3^). Different lowercase letters denote significant differences at *p* < 0.05.

As shown in Figure 6, muddy water film hole irrigation significantly increased winter wheat yield, harvest index (HI), agronomic efficiency of nitrogen (AE_N_), and irrigation water use efficiency (IWUE) compared to the CK treatment (*p* < 0.05). The T5 treatment produced the highest yield in both years, with increases of 21.34% and 24.96%, respectively, compared to the conventionally irrigated CK2 treatment. In addition, the highest HI was also observed under the T5 treatment, indicating that moderate irrigation and nitrogen application helped balance the source–sink relationship and optimize the distribution of photosynthetic products. In contrast, the lowest HI values in both years were observed in the CK1 treatment without nitrogen application, which were 25.71% and 29.50% lower than those in the T5 treatment. The highest AE_N_ was recorded under the T5 treatment, suggesting that 160 kg·ha^−1^ of nitrogen application was the threshold beyond which AE_N_ declined. IWUE was higher in all muddy water film hole irrigation treatments than in the conventionally irrigated CK2 treatment. IWUE under the T5 treatment increased by 56.40% and 57.51% in the two respective years compared to CK2. The T5 treatment achieved high AE_N_ and IWUE, demonstrating significant advantages in improving winter wheat yield and enhancing water and nitrogen use efficiency.

### 3.5. The Impact of Environmental Factors on Soil Quality and Yield of Winter Wheat

**Figure 7:**
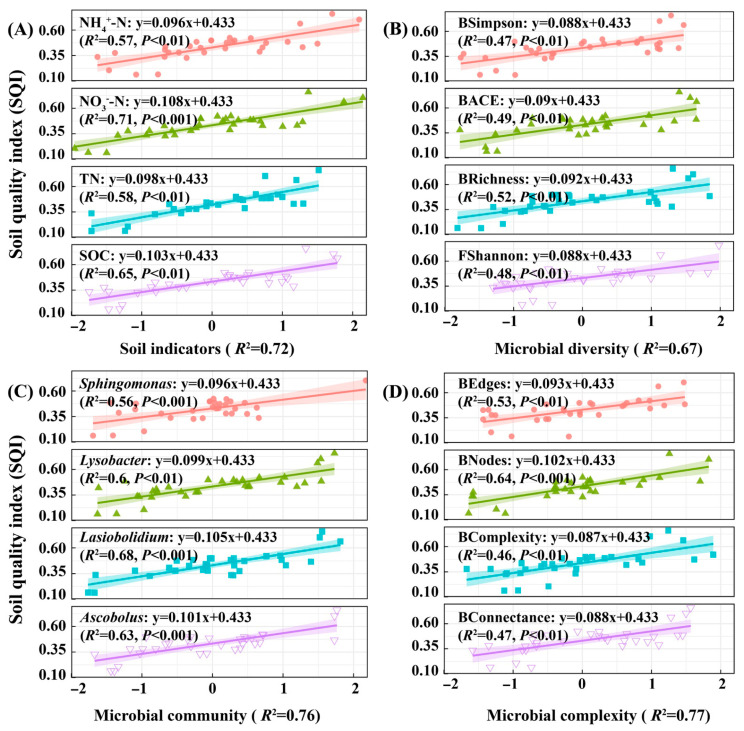
Regression analysis of environmental factors and wheat soil quality. Note: (**A**) shows the regression analysis between soil indicators (organic carbon, total nitrogen, nitrate nitrogen, ammonium nitrogen, etc.) and soil quality for winter wheat. (**B**) Regression analysis of soil microbial diversity indicators (bacteria/fungi Chao1, Shannon, Simpson, ACE, etc.) and soil quality for winter wheat. (**C**) Regression analysis of soil microbial community indicators (*Sphingomonas*, *Lysobacter*, *Lasiobolidium*, *Ascobolus*, etc.) and soil quality for winter wheat soil. (**D**) Regression analysis of co-occurrence topological network complexity indicators (nodes, edges, average degree, linkage density, etc.) and soil quality for winter wheat soil microbial networks. Due to the diversity and co−occurrence network topology complexity indicators of bacteria and fungi, which have two parts: B represents bacteria and F represents fungi, such as BSimpson representing bacterial Simpson and Fshannon representing fungal Shannon (the same applies below).

Linear regression analysis (Figure 7) indicates that soil nitrate nitrogen has the strongest explanatory power for the soil quality index (SQI), with an *R*^2^ value of 0.71. Among microbial factors, *Lysobacter*, *Lasiobolidium*, *Ascobolus*, and the number of microbial network nodes (BNodes) are also highly correlated with SQI (*R*^2^ = 0.60–0.68). Multiple linear regression analysis shows that soil physicochemical properties, microbial diversity, community composition, and network complexity are all significantly positively correlated with SQI (*R*^2^ = 0.67–0.77). Nitrate nitrogen, organic carbon, *Lasiobolidium*, and BNodes are the main influencing factors. Overall, while all indicators positively affect SQI, the contribution of microbial diversity is relatively weak. Soil nutrients and specific microbial characteristics contribute more substantially to soil quality improvement. These findings offer theoretical support for optimizing soil management practices and promoting sustainable agricultural development.

**Figure 8:**
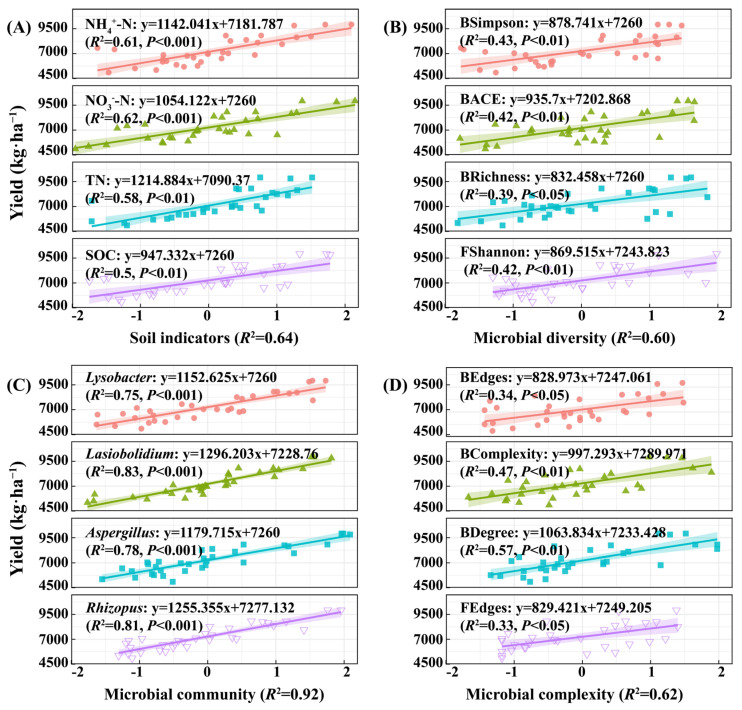
Regression analysis of environmental factors and wheat yield. Note: (**A**) shows the regression analysis between soil indicators (organic carbon, total nitrogen, nitrate nitrogen, ammonium nitrogen, etc.) and wheat yield for winter wheat. (**B**) Regression analysis of soil microbial diversity indicators (bacteria/fungi Chao1, Shannon, Simpson, ACE, etc.) and wheat yield for winter wheat. (**C**) Regression analysis of soil microbial community indicators (*Sphingomonas*, *Lysobacter*, *Lasiobolidium*, *Ascobolus*, etc.) and wheat yield for winter wheat. (**D**) Regression analysis of co−occurrence topological network complexity indicators (nodes, edges, average degree, linkage density, etc.) of soil microbial networks in winter wheat and wheat yield.

Figure 8 demonstrates that soil ammonium nitrogen and nitrate nitrogen have strong explanatory power for wheat yield, with *R*^2^ values of 0.61 and 0.63. Among microbial communities, *Lysobacter*, *Lasiobolidium*, *Aspergillus*, and *Rhizopus* have particularly significant effects on yield (*R*^2^ = 0.75−0.83). Multiple regression analysis indicates that soil physicochemical indicators, microbial diversity, community composition, and network complexity are all significantly and positively correlated with yield (*R*^2^ = 0.60−0.92). Microbial community composition has the highest explanatory power for wheat yield (*R*^2^ = 0.92). Overall, ammonium nitrogen, nitrate nitrogen, and the microorganisms are the core variables regulating yield. Microbial community structure is particularly critical for yield improvement. These results provide a theoretical basis for regulating soil microbial communities to enhance wheat yield.

### 3.6. Multidimensional Path Analysis of Environmental Factors and Yield

**Figure 9:**
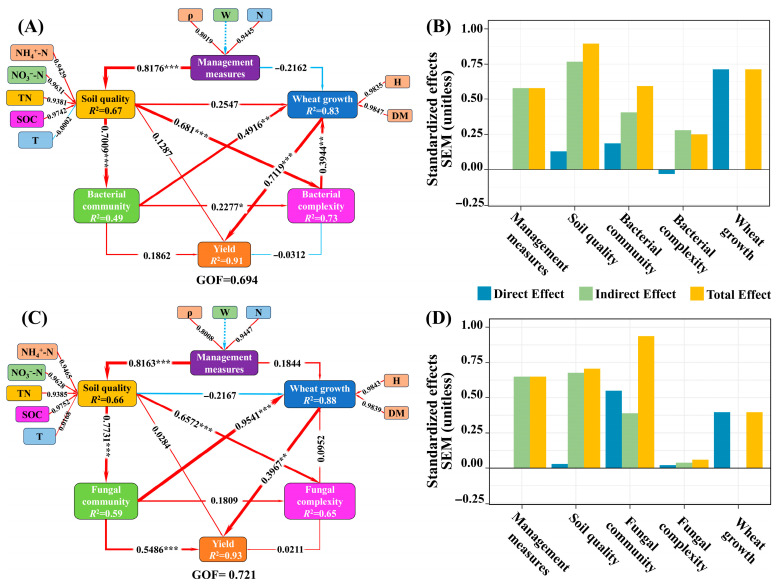
Partial least squares structural equation (PLS-SEM). Note: Figure (**A**,**C**) show the structural equation models of management measures, soil nutrients, bacterial and fungal communities, network complexity, and the impact of winter wheat growth on yield based on soil bacterial and fungal systems. The thickness of the line reflects the magnitude of the path coefficient, and the width of the arrow is proportional to the strength of the association. The red solid line and blue dashed line distinguish between positive and negative relationships. Significance symbols: * *p* < 0.05, ** *p* < 0.01, *** *p* < 0.001. Management measures include sediment concentration in muddy water (ρ), irrigation (W), and nitrogen fertilizer application (N). Soil nutrients include TN, NH_4_^+^-N, NO_3_^−^-N, SOC, SMC, pH, and T. Figure (**B**,**D**): Standardized effects modelled based on partial least squares structural equations of soil bacteria and fungi can be classified into indirect effects, direct effects, and total effects.

Results from partial least squares structural equation modeling (PLS-SEM) (Figure 9A for bacteria; Figure 9C for fungi) indicated that management practices, such as fertilization, significantly improved soil quality and were a primary driver for enhanced wheat yield. Soil quality indirectly contributed to yield by positively influencing microbial community structure and increasing network complexity. Specifically, soil quality had a significant positive effect on both bacterial and fungal community structures (path coefficients: 0.7009 and 0.7731) and promoted network complexity (path coefficients: 0.6810 for bacteria, 0.6572 for fungi). In the bacterial model, both bacterial community structure and network complexity directly enhanced wheat growth (path coefficients: 0.4916 and 0.3944, respectively), which subsequently contributed to yield indirectly Via improved plant growth (path coefficient from wheat growth to yield: 0.7119, *p* < 0.001). In contrast, the fungal model showed that fungal community structure exerted strong positive effects on both wheat growth (0.9541) and yield (0.5486), with the largest direct contribution to yield among all factors considered. Fungal network complexity did not significantly influence wheat growth.

The model demonstrated a good fit, with goodness-of-fit (GOF) values of 0.694 for bacteria and 0.721 for fungi. The *R*^2^ values for yield were 0.91 and 0.93, indicating a strong explanatory power. Total effect analysis revealed that soil quality had the greatest indirect contribution to yield, mediated by microbial communities and their network complexity, within the bacterial model (Figure 9B). In contrast, the direct effect of fungal community structure on yield was the most pronounced under the fungal model (total effect: 0.93; Figure 9D). In summary, soil quality and fungal community structure emerged as the primary factors influencing wheat yield, while bacterial community structure and complexity indirectly contributed to yield by promoting wheat growth. This study elucidated the multilevel regulatory pathway from soil quality through microorganisms and wheat growth to final yield, highlighting the critical roles of soil quality and fungal community structure in achieving high wheat productivity.

### 3.7. Comprehensive Evaluation Based on Entropy-Weighted TOPSIS Method

An integrated quotient TOPSIS analysis [50] was conducted using indicators of soil physicochemical properties, microbial diversity, community composition, and co-occurrence network complexity in winter wheat systems. The analysis produced relative proximity (*C*) values for various water and nitrogen treatments, as summarized in Table 3. Higher C values (0 < *C* < 1) indicate better performance across the evaluated indices. Among all treatments, T5 exhibited the highest *C* value (0.991), which was 2.5 times greater than that of the CK2 treatment under conventional irrigation and fertilization. These findings suggest that the T5 (ρ2W2N2) treatment is optimal for enhancing soil quality, stimulating microbial activity, and increasing winter wheat yield.

## 4. Discussion

### 4.1. The Effect of Water and Nitrogen Regulation on Soil Physical and Chemical Properties

Muddy water film hole irrigation has significant advantages in increasing soil organic carbon and nitrogen content, which will improve soil quality and microbial community structure [51]. In this experiment, the T5 treatment with moderate sediment concentration (6 kg·m^−3^), moderate irrigation volume (0.65–0.80 FC), and moderate nitrogen application rate (160 kg·ha^−1^) significantly improved the nutrient levels (TN, NH_4_^+^-N, NO_3_^−^-N, and SOC) in winter wheat soil. Especially for NH_4_^+^-N content, the T5 treatment increased by31.73% compared to CK2 control treatment. This is because the stable soil water-thermal microenvironment created by plastic film coverage promotes microbial community activity and diversity [52], enhancing soil organic matter mineralization and nitrogen transformation efficiency [53]. Additionally, the fine-grained silt introduced by moderate muddy water irrigation improved soil structure and nitrogen ion adsorption, further enhancing nutrient storage and supply capacity. Related results indicate that appropriately adjusting irrigation strategies can significantly enhance soil nitrogen availability, thereby optimizing crop growth environments [54]. Furthermore, high nitrogen application treatments (e.g., T2 and T9) resulted in a significant decrease in soil pH (6.7% and 7.22% lower than the initial value, respectively). This acidification may promote nitrification and release H^+^ [55], thereby reducing soil nutrient availability (e.g., the solubility of phosphorus and calcium) and significantly decreasing SQI [56]. This study found that muddy water sediment concentration (ρ) and nitrogen application rate (N) had a significant positive effect on SQI and TN, which is similar to the results reported by Li et al. [57], indicating that fertilizer and straw incorporation increased SQI. SQI is significantly influenced by soil physical and chemical properties, with Masto et al. [58] indicating that the primary driving variable for SQI is SOC. This study found that winter wheat soil SOC, TN, NO_3_^−^-N, and NH_4_^+^-N had a highly significant positive correlation with SQI. This is consistent with the findings of Guo et al. [59], who reported that, except for pH, which was negatively correlated with SQI, other soil properties (DON, AP, SOC, and AK) were positively correlated with SQI. In this study, the soil quality index (SQI) of winter wheat reached its highest value (0.68) under the T5 treatment, further demonstrating that film-pore irrigation with moderate muddy water sediment concentration, moderate irrigation volume, and moderate nitrogen application plays a significant role in enhancing soil comprehensive health. Therefore, moderate water and nitrogen regulation measures effectively optimize the ecological environment for winter wheat growth through water-soil-microbial interactions, providing important references for sustainable agricultural management in the middle and upper reaches of the Yellow River.

### 4.2. Soil Microbial Communities’ Response to Water Nitrogen Regulation

Different treatments of muddy water sediment concentration, irrigation volume, and nitrogen application significantly influenced the diversity and structure of the soil microbial community in winter wheat. Specifically, the soil bacterial alpha diversity indices (Chao1, ACE, and Richness) showed a trend of first increasing and then decreasing with increasing water-nitrogen treatment intensity, consistent with the findings of Shen et al. [50], who reported that soil microbial diversity in ginseng soil first increased and then decreased with increasing water-nitrogen application rates. Among all treatments, the moderate water-nitrogen treatment (T5) significantly increased bacterial community richness and evenness, with the Chao1 index increasing by 53.39% and 48.22% compared to the control treatments (CK1 and CK2), respectively. This indicates that moderate irrigation and fertilization levels provide a more suitable environment for bacterial community survival and reproduction, consistent with the findings of Muhammad et al. [60], who found that moderate nitrogen application resulted in higher soil enzyme activity, soil nutrient content, and bacterial α and β diversity compared to high nitrogen application. Additionally, the Shannon and Simpson indices for fungal communities reached their highest values in the T5 treatment, further confirming that appropriate water and nitrogen conditions significantly enhance fungal community stability and diversity [61]. However, the Chao1 and ACE indices of fungal communities reached their maximum under high nitrogen application conditions (T2), reflecting the differences between fungi and bacteria in ecological niches and environmental requirements. Fungal communities may be more sensitive to nitrogen fertilizer input than bacterial communities [62], as evidenced by their greater tendency to increase in richness under high nitrogen environments. This is similar to the findings reported by Yang et al. [63] in their study on the effects of drip irrigation nitrogen fertilizer on soil microbial diversity in spring corn, where nitrogen application had a greater impact on fungi than on bacteria.

Further analysis of the microbial community structure revealed that water nitrogen regulation significantly influenced the abundance and ecological function of dominant bacterial genera. Under optimal water-nitrogen conditions (T5 treatment), the abundance of bacteria involved in organic nitrogen mineralization (e.g., *Sphingomonas* and *Lysobacter*) and fungi closely associated with the nitrogen cycle (e.g., *Lasiobolidium* and *Ascobolus*) was significantly enhanced. This indicates that moderate water–nitrogen conditions can effectively optimize microbial community structure, enhance soil organic matter decomposition and nitrogen mineralization functions, and thereby improve soil nutrient supply capacity [64]. This result is consistent with previous studies, which concluded that appropriate irrigation and nitrogen application combinations can promote the enrichment of specific functional microbial communities and significantly enhance soil nutrient availability [60,65]. Additionally, this study revealed the differing mechanisms by which mulching and silt deposition influence microbial communities. Mulching treatment (CK1) significantly increased bacterial abundance by reducing water evaporation and stabilizing soil temperature and humidity [66]. The high-sediment concentration (ρ3) treatment, on the other hand, buffered soil diurnal temperature fluctuations through a thicker silt layer, further increasing bacterial abundance (e.g., *Sphingomonas* increased by 7.23% compared to ρ1). However, the high-sediment concentration (ρ3) treatment restricted oxygen diffusion beneath the mulch, reducing aerobic fungal activity and significantly decreasing fungal abundance. This finding was validated by the significant increase in Rhizopus abundance under low-sand conditions (T2 and T3). The co-occurrence network connectivity density of fungi was lower than that of bacteria, reflecting the greater sensitivity of fungal communities to changes in soil microenvironments [67]. The results of the nitrogen application response further confirmed this sensitivity: high nitrogen application (N3) treatment caused a significantly greater change in fungal community structure than bacterial community structure, consistent with the previously reported sensitivity of fungal communities to nutrient fluctuations [68]. In summary, this study elucidated the underlying mechanisms of microbial community structure changes under different water-nitrogen treatments. Specifically, moderate sediment concentration, moderate irrigation, and nitrogen application conditions (T5) optimize soil water-thermal environment and nutrient supply, promoting the enrichment of functional microorganisms, thereby enhancing soil nitrogen transformation and utilization efficiency, and providing important ecological support for winter wheat yield improvement.

### 4.3. The Effect of Water and Nitrogen Regulation on the Complexity of Microbial Co-Occurrence Net Works

The complexity of microbial co-occurrence networks is strongly associated with ecosystem stability. An increase in the number of nodes, edges, and connection density generally signifies more frequent interactions between microorganisms and a higher degree of ecological functional synergy [69]. This study found that soil bacterial networks exhibit greater complexity than fungal networks, evidenced by a higher number of nodes, increased connection density, and more cross-module connection points (Connectors). The proportion of Connectors in bacterial networks (6.69%) is notably higher than in fungal networks, suggesting that bacteria play a more prominent role in cross-module functional coupling and information transmission within soil ecosystems [70]. In contrast, the proportion of peripheral nodes in fungal networks reached 98.95%, indicating the strong functional modularity of fungal communities, where distinct fungal groups tend to perform specific ecological functions independently. Further analysis revealed that phyla such as *Proteobacteria*, *Actinobacteriota*, and *Bacteroidota* frequently co-occur in bacterial networks and are involved in critical ecological functions such as soil nutrient cycling and carbon-nitrogen conversion [71]. This study also identified soil nutrients (TN, SOC, NO_3_^−^-N, NH_4_^+^-N) and nitrogen application levels (N) as key factors affecting microbial network complexity. Specifically, increased NO_3_^−^-N content significantly promotes microbial community diversity and network complexity, consistent with Liu et al. [72], who found that NO_3_^−^-N is a key nutrient driving bacterial community changes. Thus, it can be inferred that nitrogen supply influences microbial interactions and ecological network structure by regulating soil microbial growth and metabolic activity [73]. A combination of moderate muddy water sediment concentration, irrigation volume, and nitrogen application rate significantly increased the number of nodes, connection density, and overall network complexity in the soil microbial network. This suggests that appropriate water–nitrogen management can optimize microbial ecological networks, enhance microbial community resilience to external disturbances, and improve ecosystem functional stability [74]. Conversely, extreme water-nitrogen conditions weaken microbial co-occurrence networks, potentially reducing the soil ecosystem’s capacity to withstand disturbances and affecting the health and stability of agricultural ecosystems [75]. Therefore, regulating water-nitrogen inputs to optimize soil microbial ecological network complexity is an effective strategy for achieving synergistic improvements in soil ecological function and productivity in winter wheat-growing regions.

### 4.4. Multidimensional Driving Mechanisms of Soil Quality and Microbial Communities on Wheat Yield

This study used multiple regression and structural equation modeling to explore the multidimensional driving mechanisms of soil nutrients, microbial community structure, and network complexity on winter wheat yield. The multiple regression analysis revealed that nitrate nitrogen (NO_3_^−^-N) and soil organic carbon (SOC) were the primary factors driving the soil quality index (SQI) (*R*^2^ = 0.71 and 0.65, respectively) (Figure 7). These factors directly enhance fertility and activate functional microorganisms, such as *Lysobacter* (with organic nitrogen mineralization capacity) and *Lasiobolidium* (promoting nitrogen cycling), accelerating organic matter decomposition, nitrogen transformation, and enhancing nutrient availability [76]. The number of bacterial network nodes (BNodes) was significantly correlated with SQI (*R*^2^ = 0.64), indicating that network stability and community interactions are key mediators of soil quality optimization. In the wheat yield pathway (Figure 8), ammonium nitrogen and nitrate nitrogen explained *R*^2^ = 0.61 and 0.62 of the variance, while microbial community composition contributed the most (*R*^2^ = 0.92). Among these, the enriched species *Lasiobolidium* and *Rhizopus* created an “amplification effect” by enhancing root-zone nitrogen and phosphorus cycling and plant absorption efficiency, thereby promoting biomass accumulation and grain formation [77].

Results from PLS-SEM (Figure 9) further elucidated the differentiated pathways of bacteria and fungi in response to management measures. A key finding was that the contribution mechanisms of the two communities were distinctly different: soil quality positively influenced bacterial community structure and network complexity, but bacteria primarily promoted yield indirectly, mediated through ‘Wheat growth’. In contrast, soil quality significantly drove fungal community structure, and the fungal community structure exerted the most powerful direct positive influence on ‘Yield’ (path coefficient 0.5486). This path differentiation reflects their fundamentally different niche strategies under water-nitrogen regulation. Bacteria, typically acting as r-strategists, are foundational for rapid nutrient mineralization [78]. These r-strategist bacterial networks (Figure 3A) are primarily responsible for providing the basic available nutrients (e.g., NH_4_^+^-N, NO_3_^−^-N) for biomass accumulation [79]. High nitrogen (N3) or extreme moisture stress (e.g., W1 or W3) tends to disproportionately stimulate the rapid proliferation of these r-strategists [80,81]. However, the ‘moderate’ water-nitrogen input and more stable aerobic microenvironment provided by the T5 treatment suppressed excessive bacterial competition and provided ideal conditions for K-strategists (fungi) to establish their efficient networks [82,83]. In contrast, fungi acted as K-strategist ‘specialized transporters and processors’ [84]. Their core role in organic matter decomposition and nitrogen release [85] is crucial for high-yield formation. Their direct path to yield can be attributed to several mechanisms: First, fungal hyphal networks act as critical ‘nutrient pipelines’ [86], actively exploring the soil matrix to transport resources (like N and P) from inaccessible micropores directly to the plant root, bypassing soil solution competition [87]. Second, as our results show, key fungi enriched in the T5 treatment (e.g., *Aspergillus* and *Rhizopus*) are highly efficient Phosphate Solubilizing Fungi (PSF). They unlock fixed soil phosphorus—a key limiting trigger for reproductive growth (grain filling) and final ‘Yield’, not just biomass [88]. Thus, the bacterial community establishes the foundational fertility for ‘growth’, while the fungal community acts as an efficient transport and processing system, providing the specific limiting resources (e.g., P) that determine final grain yield [89,90]. This explains their strong, direct statistical contribution in our model and validates the bridging function of the microbial ecosystem between soil quality and yield.

By integrating the results from both multiple regression and structural equation modeling, this study established a multi-pathway regulatory framework for the “soil–microbes–wheat yield” system. nitrate nitrogen, ammonium nitrogen, SOC, *Lysobacter*, *Lasiobolidium*, *Rhizopus*, and *Ascobolus* were identified as the key factors affecting soil quality and yield. Among these, *Ascobolus* plays a synergistic role in maintaining soil fertility and promoting yield formation by participating in organic matter decomposition and nitrogen mineralization. Optimizing soil nitrogen and carbon sources enhances the enrichment and metabolic activity of functional microorganisms, and the microbial process drives the “soil–microbe–crop” system synergistically by improving soil structure and nutrient supply chains. Specifically, in the T5 treatment (moderate sediment at 6 kg·m^−3^, moderate irrigation at 0.65–0.80 FC, and 160 kg·ha^−1^ N), both soil quality (SQI = 0.68) and microbial network stability and functional microbial community activity were enhanced, leading to a dual optimization of SQI and yield. These results validate the effectiveness of the “soil–microbial–wheat yield” multi-pathway regulatory model and offer theoretical and practical support for water and nitrogen management in the middle and upper reaches of the Yellow River and similar ecosystems.

## 5. Conclusions

This study systematically revealed that muddy water film hole irrigation, when coupled with optimized nitrogen application, significantly improves the soil environment and winter wheat yield in the middle and upper reaches of the Yellow River. We identified that a moderate combination of sediment, irrigation, and nitrogen (represented by the T5 treatment) created the optimal effect, enhancing key soil nutrients (TN, NH_4_^+^-N, NO_3_^−^-N, and SOC) and fostering a more diverse and complex soil microbial network. Structural equation modeling indicated that soil quality indirectly enhances yield by regulating microbial community structure and network complexity, with fungal community structure making the strongest direct contribution to yield. This study elucidates the multi-level regulatory mechanism of the “soil–microbes–crops” system, providing a theoretical basis and practical strategies for scientific water and nitrogen management, and sustainable yield increases in the Yellow River Basin and similar muddy water irrigation areas. The results of this study are based on data from a two-year experimental period and have a short-term effect. However, soil ecosystems, especially key indicators such as soil organic carbon accumulation and microbial community succession, typically require longer periods of time to exhibit cumulative effects and reach stable equilibrium. Therefore, conducting long-term, in situ experiments is crucial for verifying whether the observed short-term benefits are sustainable and evaluating the system’s adaptability to interannual climate change. This is an important direction for our team’s future research.

## Figures and Tables

**Table 1:** Orthogonal experimental design for winter wheat 2023–2024 and 2024–2025.

Treatments	Sediment Concentration (kg·m^−3^)	Irrigation Levels (FC)	Nitrogen Application (kg·ha^−1^)
T1	3	0.50–0.65	100
T2		0.65–0.80	220
T3		0.80–0.95	160
T4	6	0.50–0.65	220
T5		0.65–0.80	160
T6		0.80–0.95	100
T7	9	0.50–0.65	160
T8		0.65–0.80	100
T9		0.80–0.95	220
CK1	0	0.80–0.95	0
CK2		0.80–0.95	220

Note: Sediment concentration levels include ρ1 (3 kg·m^−3^), ρ2 (6 kg·m^−3^), and ρ3 (9 kg·m^−3^); irrigation levels include W1 (0.5–0.65 FC), W2 (0.65–0.80 FC), and W3 (0.80–0.95 FC); The nitrogen application levels included N1 (100 kg·ha^−1^), N2 (160 kg·ha^−1^), N3 (220 kg·ha^−1^). FC was field water holding capacity, 23.5%, The same as below.

**Table 2:** Soil microbial alpha diversity of winter wheat.

Treatments	Bacterial					Fungal				
	Chao1	Shannon	Simpson	ACE	Richness	Chao1	Shannon	Simpson	ACE	Richness
T1	1758.5 i	9.45 f	0.9953 c	1964.29 e	1821.85 f	135.71 h	2.89 g	0.75 g	184.15 f	160.14 d
T2	2301.3 b	9.79 a	0.9965 a	2314.65 b	2397.85 ab	221.37 a	4.16 b	0.89 b	225.6 a	391.27 b
T3	2082.99 d	9.61 c	0.9959 b	2078.45 d	2124.43 c	169.86 f	3.58 d	0.82 d	179.23 h	191.68 cd
T4	1822.03 h	9.55 d	0.9963 a	1813.51 g	1866.13 f	175.11 e	3.33 e	0.77 f	188.61 e	176.65 d
T5	2419.02 a	9.65 b	0.9965 a	2436.97 a	2473.96 a	203.72 b	5.26 a	0.93 a	191.58 c	528.46 a
T6	2191.86 c	9.51 e	0.9951 c	2202.92 c	2295.5 b	155.15 g	3.13 f	0.79 e	134.18 i	181.61 d
T7	1965.97 f	9.31 i	0.9956 b	1973.95 e	2037.18 cd	178.22 d	3.12 f	0.86 c	180.54 g	184.54 d
T8	1967.65 e	9.35 h	0.995 c	1755.75 h	1914.66 ef	177.29 d	3.74 c	0.78 ef	190.57 d	170.59 d
T9	1882.82 g	9.41 g	0.9963 a	1882.38 f	1995.8 de	188.03 c	4.15 b	0.86 c	195.99 b	227.37 c
CK1	1577.05 k	9.08 k	0.9942 e	1558 j	1597.48 g	84 j	2.29 h	0.56 i	96.45 k	69.91 f
CK2	1632.09 j	9.17 j	0.9946 d	1626.03 i	1670.77 g	111.23 i	2.92 g	0.72 h	122.95 j	115.87 e

Note: Alpha diversity of soil bacteria includes Chao1, Shannon, Simpson, ACE, and Richness indices. Chao1 and ACE indices are bacterial community richness indices, and Shannon and Simpson indices are community diversity indices. The data are the mean values of three parallel treatments, and different lowercase letters after the data in the same column indicate significant differences between treatments (*p* < 0.05), the same below.

**Table 3:** The entropy weight TOPSIS integrated evaluation.

Treatment	*D^+^*	*D* ^−^	*C*	Sort Results
T1	60.50	24.760	0.290	10
T2	22.531	56.132	0.714	3
T3	17.361	66.041	0.792	2
T4	44.709	40.465	0.475	6
T5	0.730	78.266	0.991	1
T6	54.375	27.763	0.338	9
T7	38.753	52.980	0.578	5
T8	46.087	33.072	0.418	7
T9	27.601	51.765	0.652	4
CK1	78.134	2.005	0.025	11
CK2	54.869	35.102	0.390	8

Note: *D^+^* is positive ideal solution distance; *D*^−^ is negative ideal solution distance; *C* is relative proximity.

## Data Availability

The datasets generated during and/or analyzed during the current study are available from the corresponding author on request.

## Supplementary Materials

The following supporting information can be downloaded at https://www.mdpi.com/article/10.3390/plants14223461/s1.
