# Supplementary material for: Water–Nitrogen Coupling Under Film Mulching Synergistically Enhances Soil Quality and Winter Wheat Yield by Restructuring Soil Microbial Co-Occurrence Networks
Source: Plants (Basel). 2025 Nov 13;14(22):3461. doi: 10.3390/plants14223461 (PMC12655626; doi:10.3390/plants14223461)
Supplement: Supplementary file 1 [file plants-14-03461-s001.zip › plants-3917033-supplementary.pdf]

Supplementary S1. Temperature and precipitation during the growth period of winter wheat in 2023–2024 and 2024–2025.

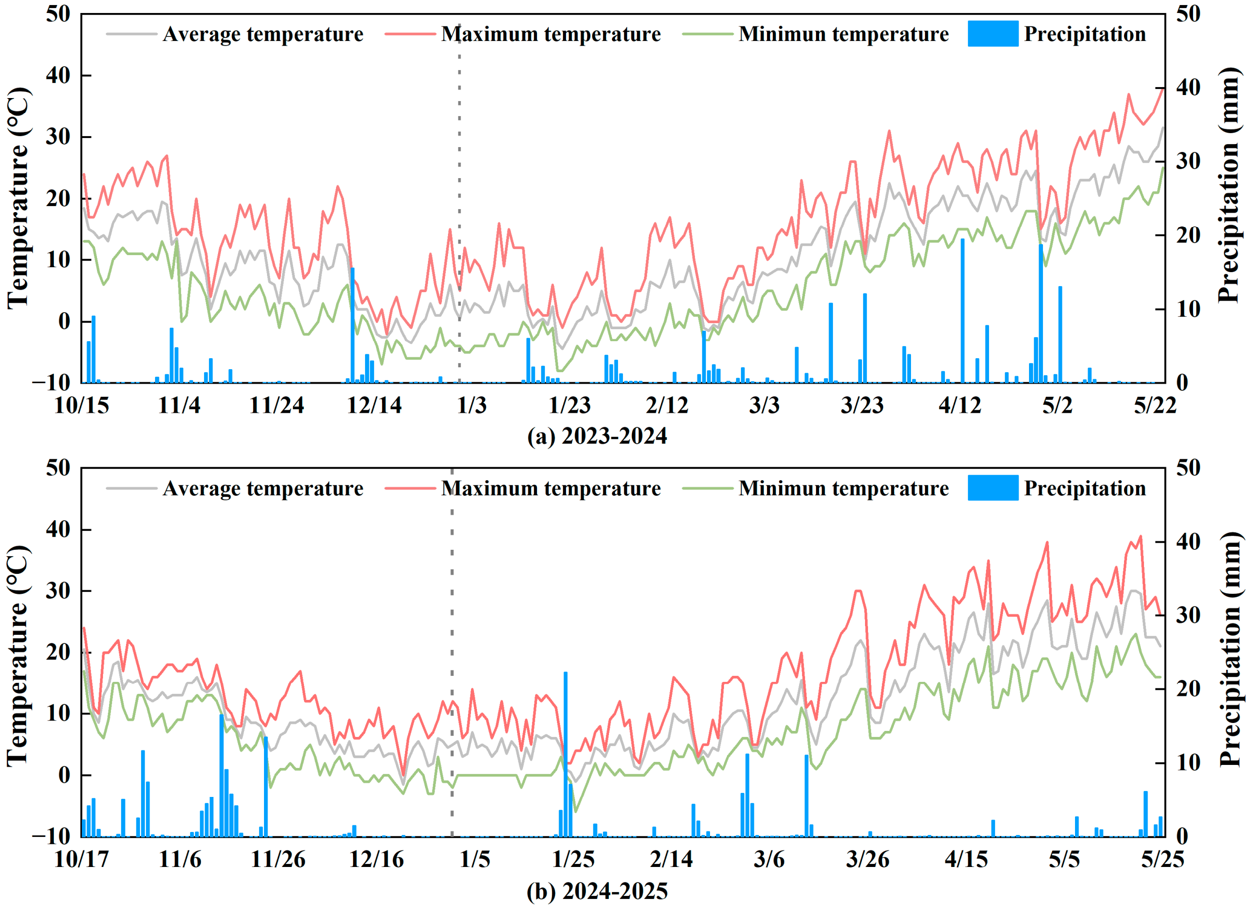

Supplementary. S2 Schematic diagram of winter wheat planting.

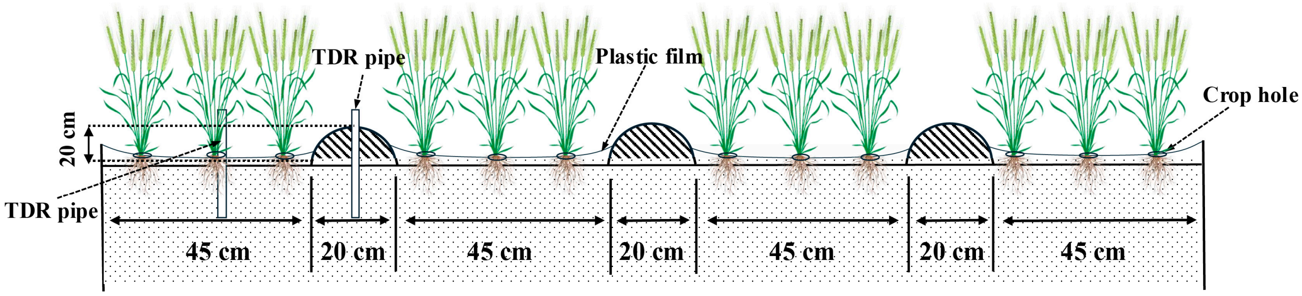

Supplementary S3. Physical and chemical properties of winter wheat soil.

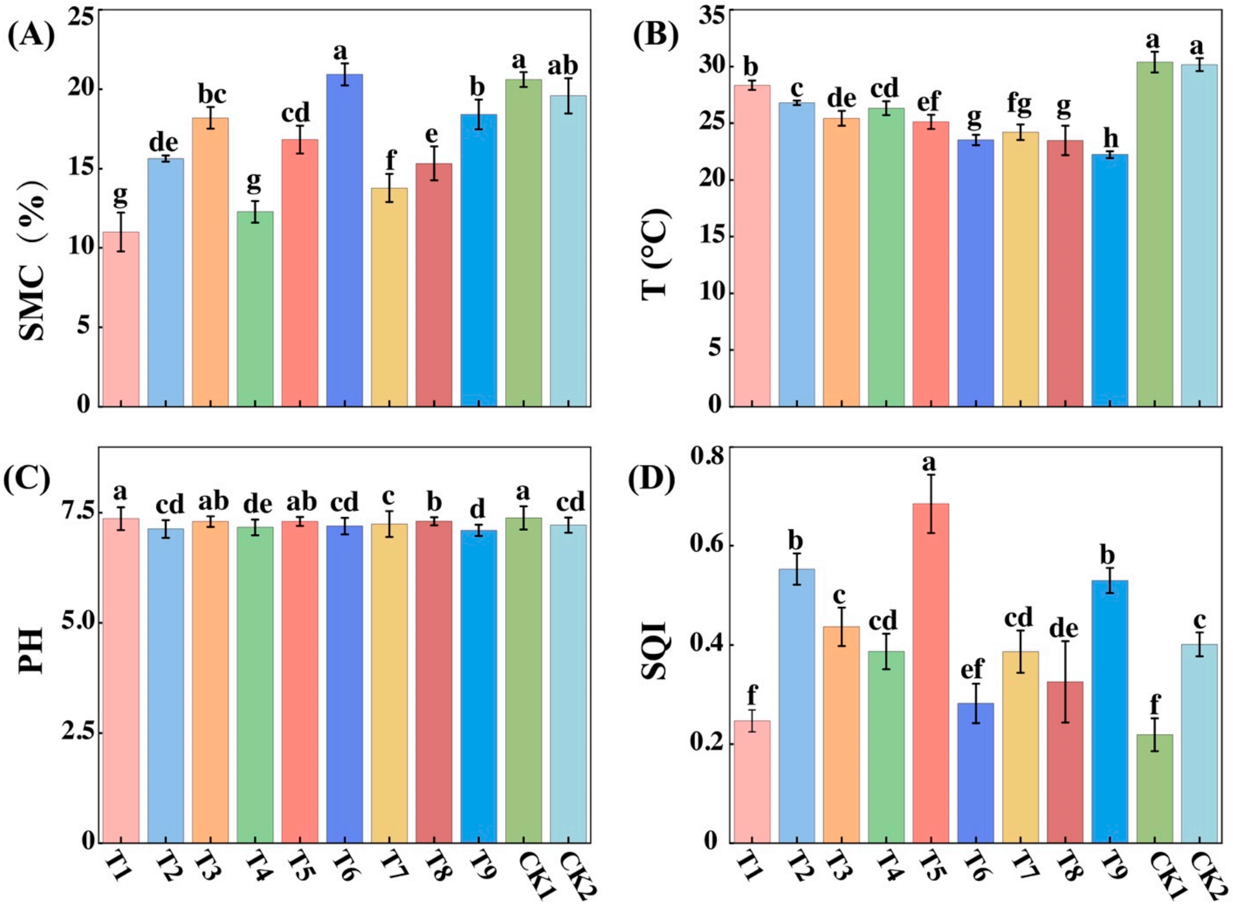

Note: Where SMC (A) denotes soil moisture content (%), T (B) denotes soil temperature °C, pH (C), and SQI (D) denotes soil quality index, with the solid line being the normal curve, p<0.05. the same below.

Supplementary. S4 Winter wheat microbial community abundance (genus level).

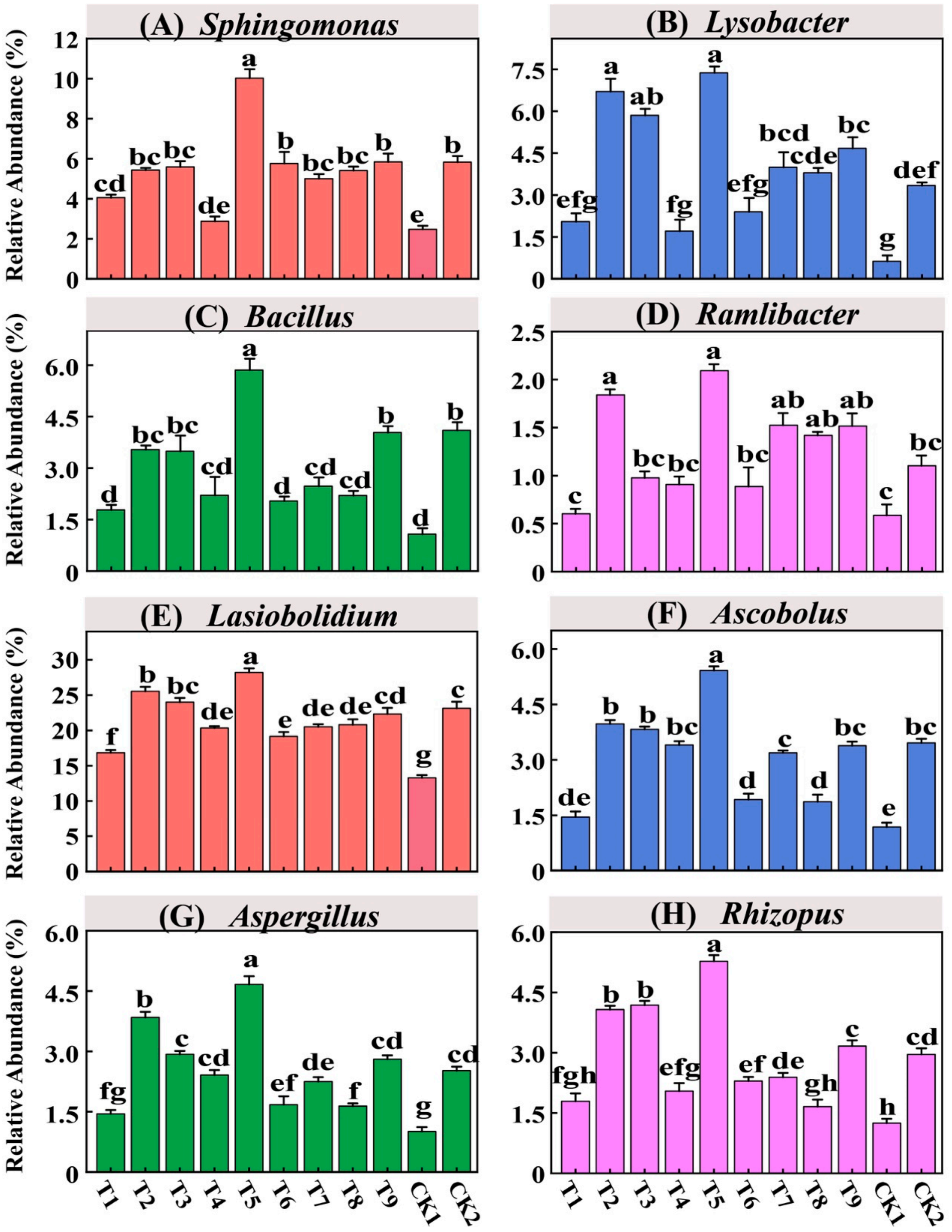

Note: The Fig (A–D) shows the top four soil bacterial genera in terms of abundance, while Fig. (E–H) shows the top four soil fungal genera in terms of abundance. Different lowercase letters denote significant differences at $p<0.05$.

Supplementary. S5 Topological parameters of soil microbial co-occurrence network.

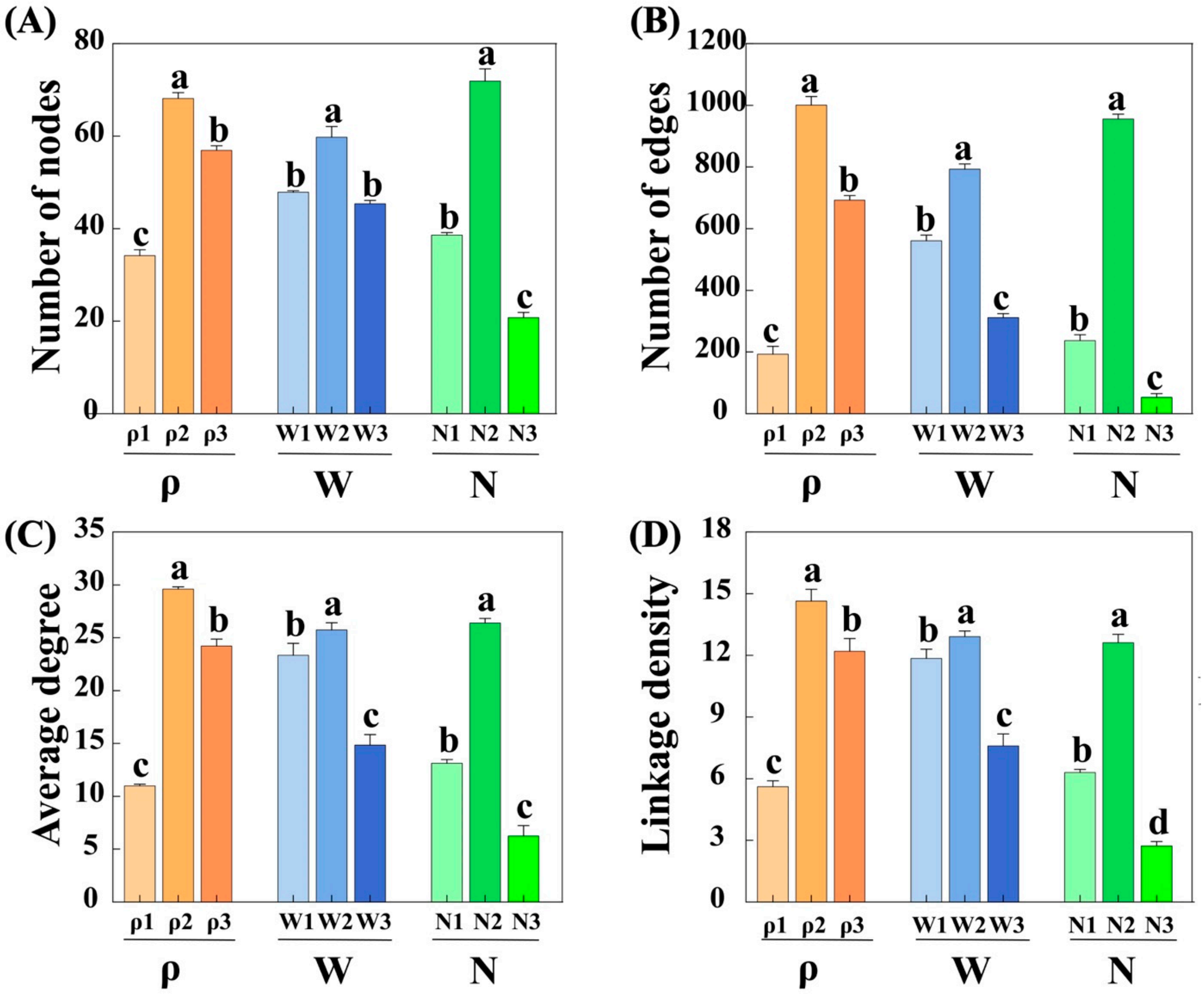

Note: (A–D) are indicators of soil microbial co-occurrence network topology, including the number of nodes (A), number of edges (B), average degree (C), and linkage density (D). In each panel of (A–D), the values of the co-occurrence networks topology are shown for different muddy water sediment concentration (ϱ1, ϱ2, ϱ3), irrigation amount (W1, W2, W3), and nitrogen application (N1, N2, N3), respectively. Different lowercase letters denote significant differences at $p<0.05$.
